# miR-17-3p Contributes to Exercise-Induced Cardiac Growth and Protects against Myocardial Ischemia-Reperfusion Injury: Erratum

**DOI:** 10.7150/thno.115473

**Published:** 2025-05-15

**Authors:** Jing Shi, Yihua Bei, Xiangqing Kong, Xiaojun Liu, Zhiyong Lei, Tianzhao Xu, Hui Wang, Qinkao Xuan, Ping Chen, Jiahong Xu, Lin Che, Hui Liu, Jiuchang Zhong, Joost PG Sluijter, Xinli Li, Anthony Rosenzweig, Junjie Xiao

**Affiliations:** 1 Department of Cardiology, The First Affiliated Hospital of Nanjing Medical University, Nanjing 210029, China;; 2 Cardiac Regeneration and Ageing Lab, School of Life Science, Shanghai University, Shanghai 200444, China.; 3 Massachusetts General Hospital Cardiovascular Division and Harvard Medical School, Boston, MA 02115, USA.; 4 Laboratory of Experimental Cardiology, University Medical Centre Utrecht, Utrecht 3508GA, The Netherlands.; 5 Department of Cardiology, Tongji Hospital, Tongji University School of Medicine, Shanghai 200065, China.; 6 State Key Laboratory of Medical Genomics & Shanghai Institute of Hypertension, Ruijin Hospital Affiliated to Shanghai Jiao Tong University School of Medicine, Shanghai 200025, China.

The authors regret that the original version of this paper unfortunately contained some incorrect representative images of immunofluorescent staining. We apologize that at the time of figure assembly, we chose representative images by mistake. We confirm that it would not affect any results and conclusions of the paper. The correct representative images for Figure 3D and Supplemental Figure 2C are shown below. Meanwhile, we would like to indicate that Figure 3B and 4A share the same β-Actin immunoblot band as an internal reference because the related immunoblots were visualized on the same membrane. The same situation was for Figure 6E and S1A.

The authors apologize for any inconvenience that these errors may have caused.

## Figures and Tables

**Figure A:**
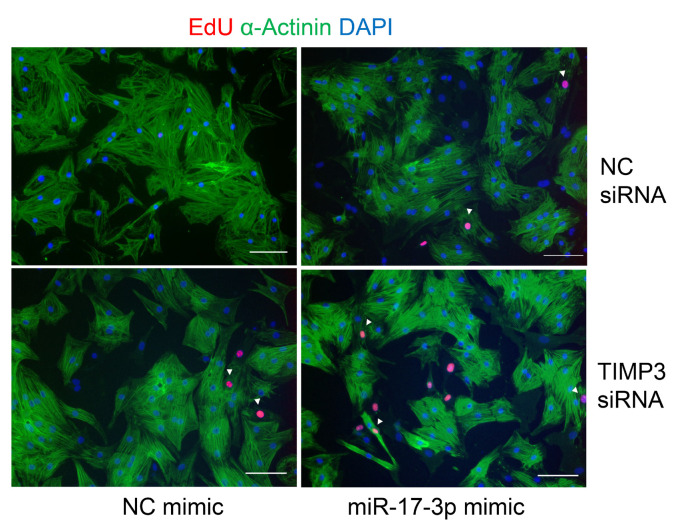
Corrected Figure 3D.

**Figure B:**
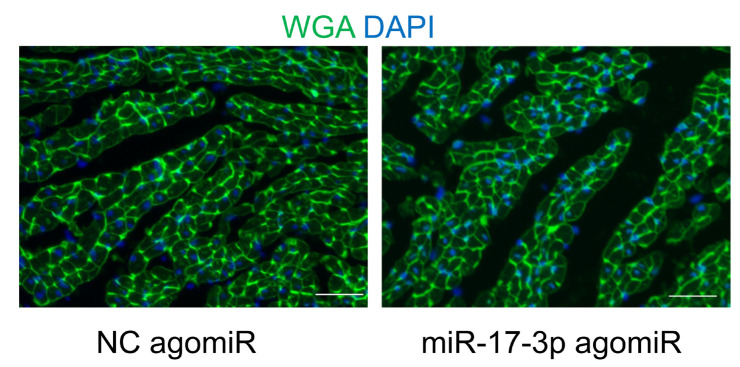
Corrected Supplemental Figure 2C.

